# Epidemiological analysis based on ORF5 of porcine reproductive and respiratory syndrome virus in China in 2019–2024

**DOI:** 10.3389/fmicb.2026.1794268

**Published:** 2026-04-10

**Authors:** Jinyong Zhang, Xiaoxiao Wei, Leilei Sun, Chen Yang, Saisai Wang, Wenbing Wang, Yue Wang, Xinhua Xu, Shengzhi Ren, Houquan Jiang, Yemou Chen, Jiafu Zhang, Canxin Deng, Congxia Wang, Ranran Lai, Lujie Bian, Lili Wu, Cuicui Jiao, Yuling Tao

**Affiliations:** 1 Shandong Engineering Research Center of Pig and Poultry Health Breeding and Important Infectious Disease Purification, Shandong New Hope Liuhe Group Co., Ltd., Qingdao, Shandong, China; 2 Key Laboratory of Digital Intelligent Breeding Technological Innovation for Swine and Poultry, Ministry of Agriculture and Rural Affairs, New Hope Liuhe Co., Ltd., Chengdu, Sichuan, China; 3 Shandong New Hope Liuhe Agriculture and Animal Husbandry Technology Co., Ltd. (NHLH Academy of Swine Research), Dezhou, China; 4 China Agriculture Research System-Yangling Comprehensive Test Station (Yangling Besun Agricultural Industry Group Corporation Co., Ltd.), Xianyang, China; 5 Immunology Laboratory, Basic Medical College, Jilin Medical University, Jilin, China

**Keywords:** epidemiological analysis, lineages, molecular epidemiology, ORF5, PRRSV

## Abstract

**Introduction:**

Porcine reproductive and respiratory syndrome virus (PRRSV) was first reported in pigs in China in 1996. At present, multiple PRRSV lineages and sub-lineages are circulating in Chinese swine herds.

**Methods:**

In this study, PRRSV ORF5 sequences collected from 2019 to 2024 were analyzed to investigate their spatiotemporal distribution, molecular evolution, and major amino acid mutations.

**Results:**

A total of 674 sequences were obtained from 25 provinces between 2019 and 2024, and their geographical distribution was generally consistent with the major pig-producing regions in China. Phylogenetic analysis revealed that these PRRSV ORF5 sequences mainly belonged to lineage 1 (L1A, L1C), L3, L5 (L5A), and L8 (L8E). From 2019 to 2024, the proportion of L1C initially increased, followed by a decrease and a subsequent rebound, while L1C remained the predominant sub lineage. The proportion of L1A showed a relative increase, whereas L3 was detected at very low levels. Meanwhile, L5A and L8E exhibited a downward trend. Amino acid mutations were observed in the signal peptide, neutralizing epitopes, and T-cell epitopes among different sub-lineages. Some mutations were variable, while others were sub-lineage-specific. Notably, partial L1C strains exhibited an amino acid deletion at position 34, which may affect the viral immune evasion mechanisms. Nucleotide and amino acid identities initially decreased and then increased, with the most pronounced divergence observed in 2021.

**Discussion:**

Continuous monitoring of the molecular epidemiology of PRRSV is essential and will provide a scientific basis for the further prevention and control of PRRSV in China.

## 1 Introduction

Porcine reproductive and respiratory syndrome (PRRS) is one of the most economically important diseases affecting the global pig industry, caused by porcine reproductive and respiratory syndrome virus (PRRSV) (Goyal, 1993; Kappes and Faaberg, 2015). The major clinical manifestations in PRRSV-infected pigs include respiratory distress, reproductive failure, elevated mortality in growing pigs, and decreased production efficiency (Li et al., 2022). PRRSV is an enveloped, single-stranded positive-sense RNA virus that belongs to the genus *Betaarterivirus*, family *Arteriviridae* (King et al., 2018; Li et al., 2024). The viral genome of PRRSV is approximately 15 kb, containing a 5′ cap structure, a 5′ untranslated region (UTR), 10 open reading frames (ORFs), a 3′ UTR and a 3′ poly (A) tail (Qiu et al., 2025). ORF1a and ORF1b encode 16 non-structural proteins (nsp1α, nsp1β, nsp2-6, nsp2TF, nsp2N, nsp7a, nsp7b, and nsp8-12), while ORF2 to ORF7 encode 8 structural proteins (GP2, GP3, GP4, GP5, GP5a, E, M, and N) (Albina, 1997; Su et al., 2019; Huang et al., 2015). PRRSV can be divided into two genotypes: Type 1 (PRRSV-1, consisting primarily of European strains and typified by Lelystad virus) and Type 2 (PRRSV-2, consisting mainly of North American strains and typified by VR-2332), which share approximately 50–70% nucleotide sequence identity (Darwich et al., 2011; Sun et al., 2023). The genome of PRRSV-2 evolves continuously and rapidly, with a nucleotide substitution rate estimated at approximately 1.55 × 10^−3^ to 9.8 × 10^−2^ substitutions per site per year (Li et al., 2024; Yim-Im et al., 2023; Zhang et al., 2022).

According to the ORF5, PRRSV-2 can be classified into 11 genetic lineages (L1–L11) and 21 sub-lineages (L1A–L1F, L1H–L1J, L5A–L5B, L8A–L8E, and L9A–L9E) (Yim-Im et al., 2023; Zhou et al., 2022). To date, lineages 1, 3, 5, and 8 have been circulating in mainland China (Zhou et al., 2022). CH-1a, the first PRRSV-2 strain reported in China, belongs to lineage 8 (L8); subsequently, strain BJ-4 was isolated in China in 1997 and classified into lineage 5 (L5) (Jian et al., 2025). In 2006, a highly virulent PRRSV variant designated highly pathogenic PRRSV (HP-PRRSV) emerged and was also assigned to lineage 8 (L8) (Tian et al., 2007). The QYYZ strain, which belongs to lineage 3 (L3), was identified in mainland China in 2010 (Sun et al., 2019). Since 2014, NADC30-like PRRSV has become a major epidemic strain in China; in 2018, NADC34-like PRRSV was first reported in China and has gradually become the dominant circulating strain (Zhao et al., 2022; Tian, 2017). Both NADC30-like and NADC34-like PRRSV isolates belong to lineage 1 (L1).

On August 3, 2018, the World Organization for Animal Health (OIE) reported the first case of African swine fever virus (ASFV) infection in Shenyang, Liaoning Province, China (Zhou et al., 2018). From that point onward, prevention and control strategies against swine diseases have been significantly strengthened in Chinese pig farms, including increased disinfection frequency, strict on-farm biosecurity measures, and improved farm management practices. Herein, we propose a scientific hypothesis that these enhanced biosecurity and prevention strategies may potentially alter the epidemic trend of PRRSV in China, though this inference has not been experimentally verified. In the present study, ORF5 gene sequences collected from 2019 to 2024 were used to analyze the molecular epidemiology of PRRSV in China, providing a baseline for subsequent validation of the above hypothesis.

## 2 Materials and methods

### 2.1 Sample collection and genome sequencing

From 2019 to 2024, samples for PRRSV detection and sequencing were collected across most provinces in China, with the sampling sources comprising 60% large-scale breeding enterprises and 40% small and medium-sized farms. The sample types included tissues (lung and lymph nodes), serum, aborted fetuses (or placentas), oral fluids, nasal swabs, and pharyngeal swabs.

The primers used for sequencing were as follows: ORF5-F: GGCAATGTGTCAGGCATCGTGGCTG; ORF5-R: GGCCAGAAT GTACTTGCGGCCTAGC (The product size is 1,116 bp).

### 2.2 Homology, phylogenetic and molecular evolutionary analyses

59 PRRSV ORF5 sequences were downloaded from Genbank^1^ as reference sequences, and their classification was performed with reference to the report by Wannarat Yim-Im et al. (Supplementary Table S1) (Yim-Im et al., 2023). The nucleotide and amino acid homology as well as mutation characteristics of PRRSV ORF5 were analyzed using MegAlign software (DNASTAR Lasergene v7.1). PRRSV ORF5 sequences were aligned via MEGA 7.0, followed by the construction of a maximum likelihood phylogenetic tree with 1,000 bootstrap replicates using the same software for phylogenetic analysis (Tamura et al., 2011). Based on the aforementioned methods, we analyzed the classification of all sequences and investigated the temporal and spatial distribution prevalence of different PRRSV lineages and sub-lineages. The online tool ITOL^2^ was employed for the annotation of the constructed phylogenetic tree (Letunic and Bork, 2024).

## 3 Results

### 3.1 Geographical and temporal distribution of samples

**Figure 1:**
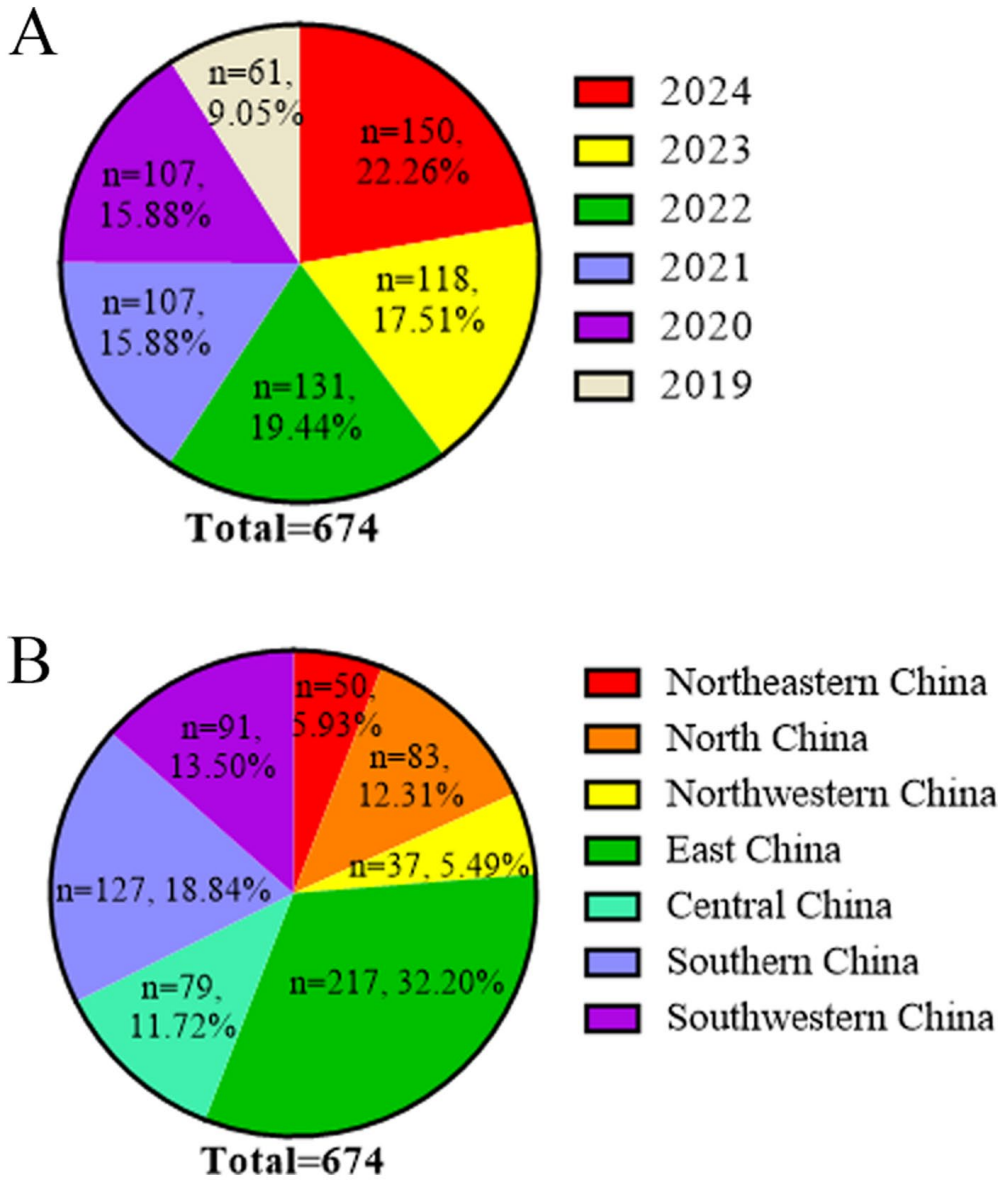
Temporal and regional distribution of PRRSV ORF5 sequences. **(A)** Annual number of PRRSV ORF5 sequences collected from 2019 to 2024. **(B)** Regional distribution of PRRSV ORF5 sequences in China.

A total of 674 PRRSV ORF5 sequences collected from 25 provinces between 2019 and 2024 were included in this study (Supplementary Table S2). Among these 674 sequences, the number of isolates exhibited an overall increasing trend over the years: 2019 (*n* = 61, 9.05%), 2020 (*n* = 107, 15.88%), 2021 (*n* = 107, 15.88%), 2022 (*n* = 131, 19.44%), 2023 (*n* = 118, 17.15%), and 2024 (*n* = 150, 22.26%) (Figure 1A). According to regional distribution, the sequences were distributed as follows: East China (*n* = 219, 32.20%), Southern China (*n* = 127, 18.84%), Southwestern China (*n* = 91, 13.50%), North China (*n* = 83, 12.31%), Central China (*n* = 79, 11.72%), Northeastern China (*n* = 40, 5.93%), and Northwestern China (*n* = 37, 5.49%) (Figure 1B). The sequence distribution was consistent with the regional density of pig populations in China, with a higher number of sequences obtained from provinces with larger swine industries, including Shandong, Guangxi, Guangdong, Sichuan, and Hebei (Table 1).

**Table 1:** Distribution of PRRSV ORF5 sequences across provinces during 2019–2024.

Region	Province	2019	2020	2021	2022	2023	2024
Northeastern China	Heilongjiang (HL)	0	0	3	4	2	2
	Jilin (JL)*	0	0	0	0	0	0
	Liaoning (LN)	1	6	3	8	5	6
North China	Beijing (BJ)	0	0	0	0	1	1
	Tianjin (TJ)	0	2	2	3	6	3
	Hebei (HE)	3	5	6	5	5	10
	Shanxi (SX)	0	4	5	2	2	3
	Inner Mongolia (NM)	0	2	4	4	3	2
Northwestern China	Shaanxi (SN)	2	1	4	2	4	6
	Gansu (GS)	0	1	3	6	4	4
	Ningxia (NX)*	0	0	0	0	0	0
	Qinghai (QH)*	0	0	0	0	0	0
	Xinjiang (XJ)*	0	0	0	0	0	0
East China	Shanghai (SH)*	0	0	0	0	0	0
	Jiangsu (JS)	9	6	3	5	3	4
	Zhejiang (ZJ)	1	1	4	5	6	7
	Anhui (AH)	3	4	5	3	5	7
	Fujian (FJ)	0	0	1	1	1	3
	Jiangxi (JX)	4	6	3	7	5	2
	Shandong (SD)	7	18	19	21	16	22
	Taiwan (TW)*	0	0	0	0	0	0
Central China	Hubei (HB)	1	5	3	4	5	9
	Henan (HA)	2	10	6	3	5	5
	Hunan (HN)	4	3	1	3	8	2
Southern China	Guangdong (GD)	7	4	6	11	7	10
	Guangxi (GX)	9	14	11	7	9	13
	Hainan (HI)	3	1	2	3	3	7
	Hong Kong (HK)*	0	0	0	0	0	0
	Macao (MO)*	0	0	0	0	0	0
Southwestern China	Sichuan (SC)	3	5	9	11	8	9
	Chongqing (CQ)	2	5	1	2	0	3
	Guizhou (GZ)	0	4	3	8	5	10
	Yunnan (YN)	0	0	0	3	0	0
	Xizang (XZ)*	0	0	0	0	0	0

*Indicates that the provinces with no samples were collected.

### 3.2 PRRSV genetic classification based on ORF5 sequences

**Figure 2:**
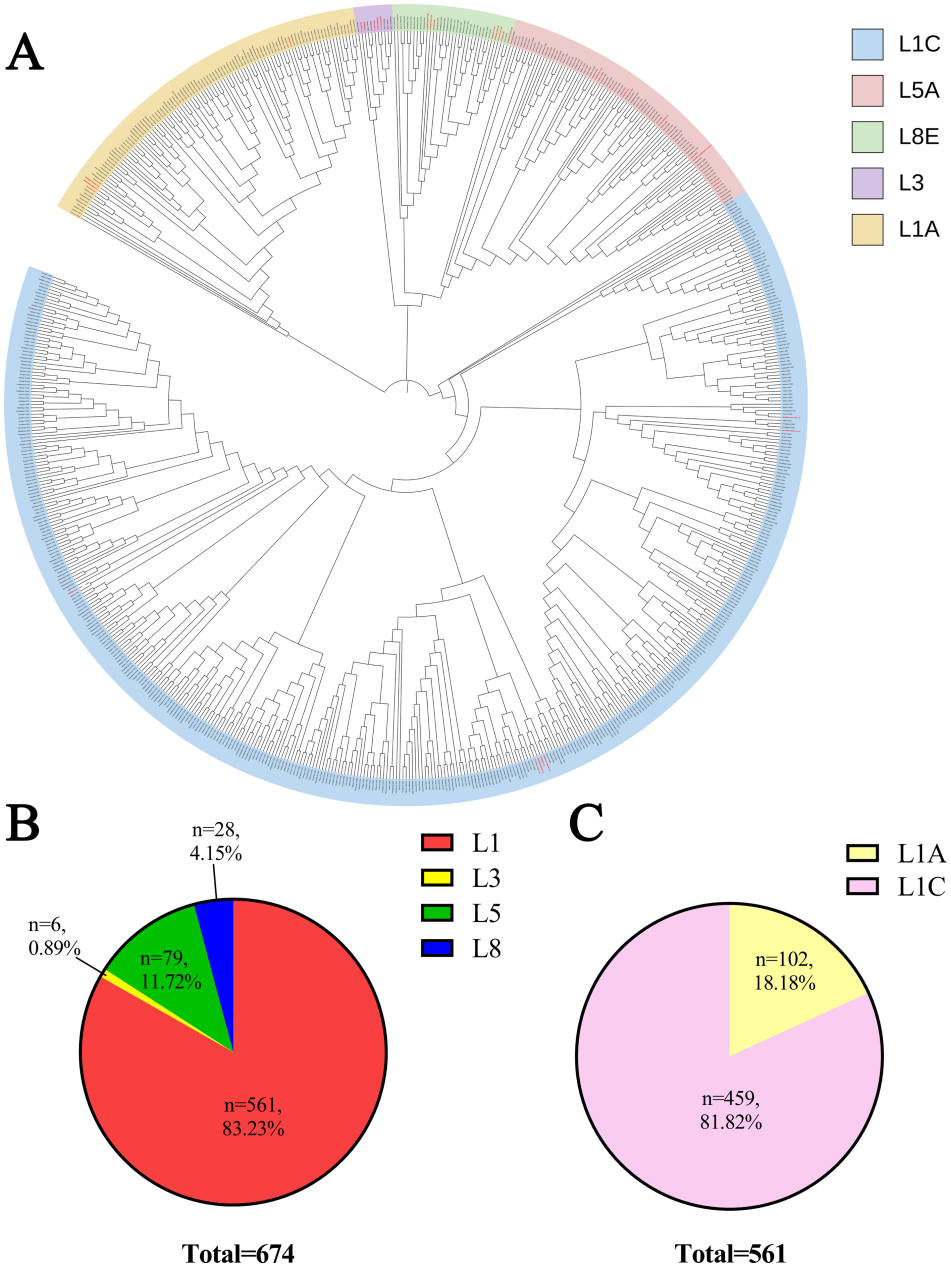
Classification and lineage/sub-lineage distribution of PRRSV ORF5 during 2019–2024. **(A)** Classification of PRRSV ORF5 sequences collected in China during 2019–2024. Based on reference sequences, all sequences were assigned to five sub-lineages: L1A, L1C, L3, L5A, and L8E. Red font indicates reference sequences. **(B)** Proportional analysis of lineages L1, L3, L5, and L8. **(C)** Proportions of sub-lineages L1A and L1C within lineage L1.

Genetic analysis based on PRRSV ORF5 sequences showed that the 674 sequences were mainly clustered into four lineages: L1, L3, L5, and L8. According to the reference sequences, these isolates were further assigned to five sub-lineages, namely L1A, L1C, L3, L5A, and L8E (Figure 2A). Of the 674 sequences, 561 (83.23%) belonged to lineage L1, 6 (0.89%) to L3, 79 (11.72%) to L5, and 28 (4.15%) to L8. L1 was further divided into sub-lineages L1A and L1C, with 102 (18.18%) and 459 (81.82%) sequences, respectively (Figure 2B).

### 3.3 Geographical distribution of PRRSV lineages in China

**Figure 3:**
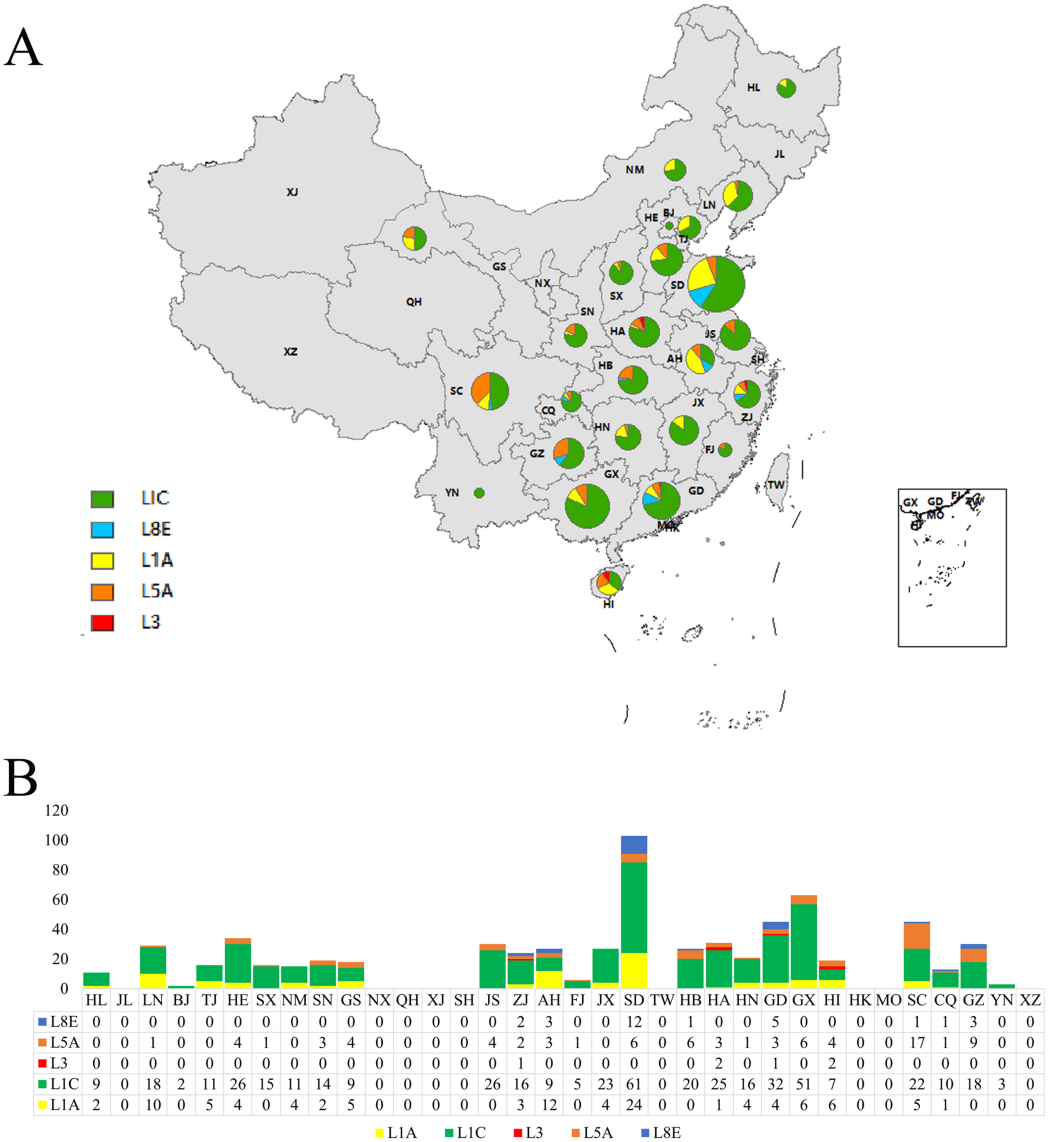
Sub-lineage distribution of PRRSV ORF5 sequences across provinces in China, 2019–2024. **(A)** Proportions of PRRSV ORF5 sub-lineages in each province shown by pie charts. **(B)** Numbers of PRRSV ORF5 sub-lineage sequences in each province shown by bar chart.

From 2019 to 2024, a total of 674 PRRSV ORF5 sequences were collected from 25 provinces. Phylogenetic and genetic evolutionary analysis revealed that these sequences could be classified into lineages L1 (including sub-lineages L1A and L1C), L3, L5 (sub-lineage L5A), and L8 (sub-lineage L8E). L1C was detected across all 25 provinces, and its proportion exceeded 50% in 22 provinces (excluding Anhui, Sichuan, and Hainan). L1A was identified in 17 provinces, with its proportion accounting for less than 30% in most regions. L3 was only detected in Zhejiang, Henan, Guangdong, and Hainan. L5 was present in 19 provinces, representing no more than 20% of sequences in most provinces; however, it accounted for approximately 37.78% in Sichuan. L8E was detected in 8 provinces, with a relatively low prevalence (Figures 3A,B).

### 3.4 Temporal distribution of PRRSV lineages in China

**Figure 4:**
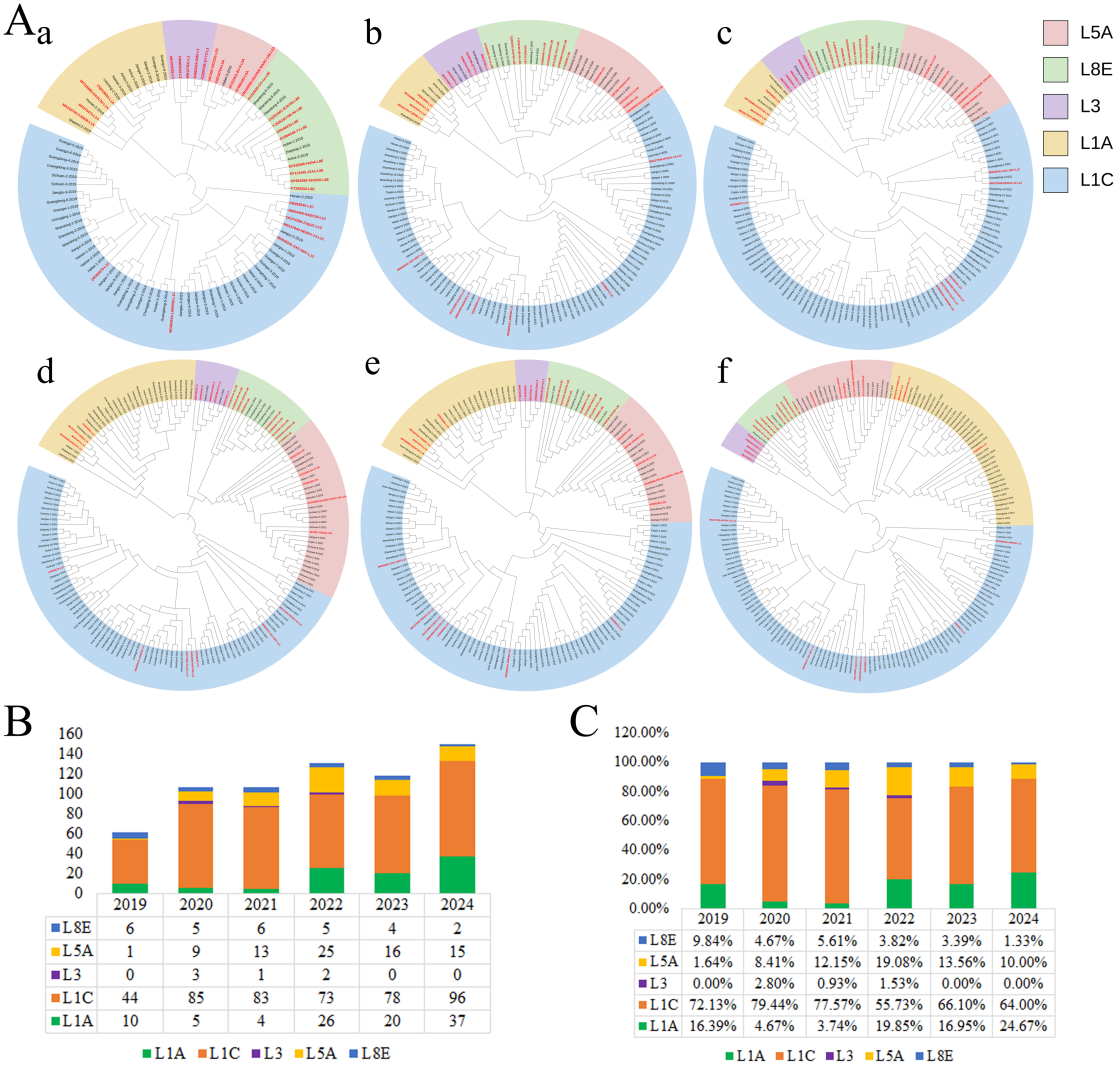
Classification and sub-lineage distribution of PRRSV ORF5 by year during 2019–2024. **(A)** a–f represent phylogenetic analyses for 2019, 2020, 2021, 2022, 2023, and 2024, respectively. Red font indicates reference sequences. **(B)** Numbers of PRRSV ORF5 sequences for each sub-lineage from 2019 to 2024. **(C)** Proportional distribution of each sub-lineage from 2019 to 2024.

From 2019 to 2024, sequences from each year were subjected to independent evolutionary analysis (Figure 4A). Based on the results shown in Figure 4A, the abundances of L1A, L1C, L3, L5A, and L8E were statistically quantified annually (Figure 4B). The detection rate of L1A was 16.39% in 2019, 4.67% in 2020, 3.74% in 2021, 19.85% in 2022, 16.95% in 2023, and 24.67% in 2024. It declined sharply in 2020 and 2021, reaching its highest detection rate in 2024. L1C exhibited an initial increase, followed by a decrease and a subsequent rise, peaking in 2020. After 2020, its overall detection rate trended downward: 72.13% in 2019, 79.44% in 2020, 77.57% in 2021, 55.73% in 2022, 66.10% in 2023, and 64.00% in 2024. L3 was detected only in 2020 (2.80%), 2021 (0.93%), and 2022 (1.53%), with relatively low proportions. L5A displayed an initial increase followed by a decrease. Its detection rate was only 1.64% in 2019, peaked at 19.08% in 2022, and stood at 10.00% in 2024. For L8E, the detection rate remained below 10.00% and showed a continuous downward trend from 2019 onward, falling to just 1.33% in 2024 (Figure 4C).

### 3.5 Results of homology analysis

The nucleotide and amino acid homology of PRRSV ORF5 was analyzed using the MegAlign software (DNASTAR Lasergene v7.1). As shown in Table 2, comparison of 674 PRRSV ORF5 sequences revealed that nucleotide sequence identities ranged from 75.3 to 100%, while amino acid sequence identities ranged from 67.7 to 100%. From 2019 to 2021, the sequence identities of PRRSV ORF5 gradually decreased, followed by a gradual increase from 2022 to 2024. Both nucleotide and amino acid similarities reached their highest levels in 2024. The lowest similarities were observed in 2021, with nucleotide identities ranging from 76 to 100% and amino acid identities ranging from 68 to 100%. In 2024, nucleotide identities ranged from 81.5 to 100% and amino acid identities from 81.1 to 100%, representing the highest homology across the study period. We further performed homology analysis among ORF5 sequences within the same sub-lineages. The greatest divergence was detected in L1C, whereas the smallest divergence was found in L5A (Table 3).

**Table 2:** Homology of nucleotide and amino acid sequences from 2019 to 2024.

Year	Nucleotide (%)	Amino acid (%)
2019	80.3–100	79–100
2020	78.8–99.7	78–100
2021	76–100	68–100
2022	79.8–100	78.1–100
2023	81.7–100	79–100
2024	81.5–100	81.1–100
2019–2024	75.3–100	67.7–100

**Table 3:** Intra-sub-lineage homology of the detected strains.

Sub lineage	Nucleotide (%)	Amino acid (%)
L1A	83.1–100	81.6–100
L1C	79.5–100	80.1–100
L3	85.4–98.7	84.6–98.5
L5A	89.4–100	93.1–100
L8E	88.1–100	83.1–100

### 3.6 Characteristic mutations among different sub lineages

**Figure 5:**
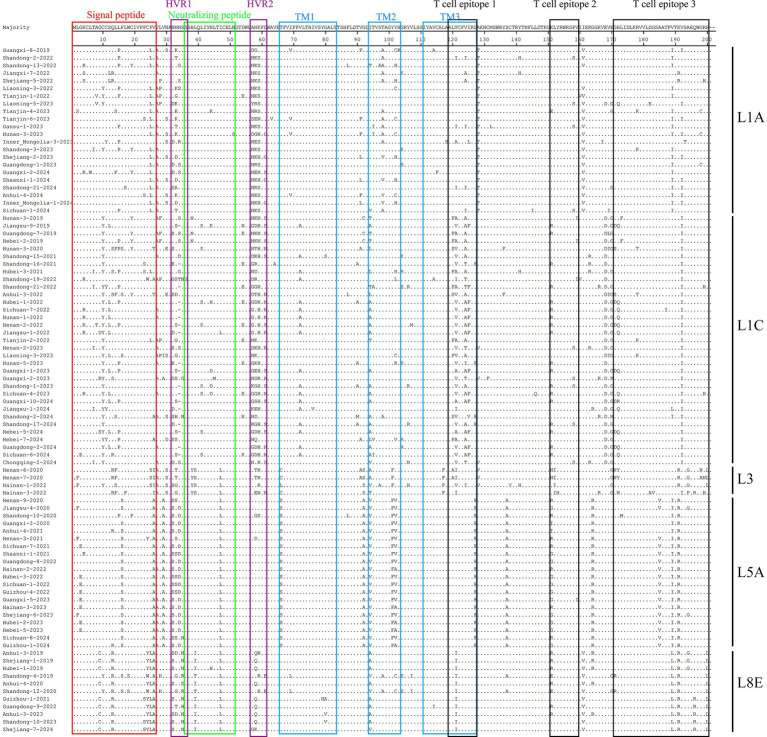
Amino acid sequence analysis of PRRSV GP5. Red box: Signal peptide; purple box: hypervariable region (HVR); green box: main neutralizing epitope; blue box: transmembrane region; black box: T cell epitope.

To investigate amino acid differences among L1A, L1C, L3, L5A, and L8E, amino acid mutations were analyzed using MegAlign software. The results revealed numerous amino acid mutations both between and within sub-lineages. As shown in Table 4 and Figure 5, some sequences in L1C exhibited a deletion at amino acid position 34. More amino acid mutations were observed in L1A and L1C, and mutation frequencies tended to be higher in sub-lineages containing more strains. Mutations at the same amino acid site varied across different sub-lineages. For instance, position 58 changed from N to K/E/G in L1A, to K/R/D/G in L1C, to T in L3, and to Q in L8E. Similarly, multiple substitutions occurred at individual sites within single lineages: in L1A, 58 N → K/E/G, 59 K → S/N/H, and 61D → G/S; in L1C, 58 N → K/R/D/G, 121 T → I/A/V, and 124 V → I/A/T. In general, more complex mutations at individual amino acid sites were detected in sub-lineages with larger numbers of strains. In addition, several sub-lineage-specific mutations were identified, including 69I → V in L1A, 72 V → A in L1C, 38H → Y and 29 V → A in L5A, and 9G → C in L8E. Multiple amino acid mutation sites were located in the signal peptide, transmembrane regions, neutralizing epitopes, and T-cell epitopes. These mutations may alter viral infection characteristics and contribute to phenomena such as immune evasion.

**Table 4:** Summarize amino acid mutation sites.

Sub lineage	Amino acid mutation	Amino acids deletion
L1A	K4R/S, C10Y, Q13R, L15P, F25L, V27A, N32D/S, A57N/D, N58K/E/G, K59S/N/H, D61G/S, I69V, T98A/V, Y102C/H, T121I/A, V124I/A, A128T, P158S, I161V, E170G, H172Q, V189I, K191R, V192I	–
L1C	K4R, C10Y, Q13S, L15P, F16S, F25L, V27A, N32D/S, H38N, L41S, A57N/G, N58K/R/D/G, K59N/H, D61N/S, V72A, G92C, I94T/A/L, T121I/A/V, V124I/A/T, L127F, A128V, K151R, P158S, E168D, E170G, H172Q, V189I/L, V192I	N34del
L3	Q13R, F25S, V27A, N32S, H38Y, L39S, I47L, N58T, K59N/R, T66S, G92S, I94T/A/V, Y101F, L117F, T121I/V, A128V, K151N, L152I, E170G, H172Y/N, K191R, V192I, P200L	–
L5A	G3E, F16S, V27A, V29A, N32S, N34D, I47L, T66C/L, G92A, I94V, Y101F, Y102A/V, L127F, S137A, K151R/G/I, G164R, A185V, V189I, K191R	–
L8E	G9C, C10Y, Q13R, F16S, F25L, N32S, L39I/T I47L, N58Q, K59N/R, D61E, I94A, T121I, K151R/E, I161V, G164R, E170G, V189L, K191R, P200L	–

## 4 Discussion

PRRSV has been circulating in China for nearly 30 years. This virus exhibits high genetic diversity, frequent mutation, and frequent recombination events, leading to continuous changes in virulence and causing substantial economic losses to the swine industry in China (Tian et al., 2025). The GP5 protein contains major antigenic epitopes associated with viral neutralization, as well as functional motifs including signal peptides, transmembrane regions, antigenic determinants, and glycosylation sites. For these reasons, GP5 is frequently used as a target antigen for the development of novel vaccines (Thuy et al., 2013; Liu and Chen, 2024). Moreover, due to its high genetic variability, GP5 is also widely employed for phylogenetic analysis (Song et al., 2020).

To resolve inconsistencies and reduce discrepancies in PRRSV-2 lineage classification among different studies, Wesley R. D. and colleagues revised the lineage classification system for PRRSV-2 based on ORF5 sequences, expanding the number of lineages from 9 to 11 (Tian et al., 2025; Wesley et al., 1998). According to previous studies, PRRSV-2 was first detected in China as early as 1991 and was classified as L3. L5 was identified in 1992, and the prototype strain CH-1a, belonging to L8, was first reported in China in 1996. From 1997 to 2001, L3 became the dominant strain, while L5 was also detected. During 2002–2005, L3, L5, and L8 were co-circulating, with L8 emerging as a potential dominant lineage. From 2006 to 2015, L8 was the predominant strain. NADC30-like PRRSV was reported in China in 2012, after which lineages L1, L3, L5, and L8 became co-endemic in Chinese pig herds. From 2018 to the present, L1 has been the dominant lineage (Tian et al., 2025; Zhou et al., 2024; Guo et al., 2018; Lv et al., 2024; Zhao et al., 2022; Luo et al., 2023; Ouyang et al., 2024; Zhang et al., 2024; Sun et al., 2024). In the present study, a total of 674 PRRSV ORF5 sequences, obtained from samples collected across 25 provinces of China from 2019 to 2024 (encompassing 60% large-scale breeding enterprises and 40% small and medium-sized farms), were subjected to comprehensive analysis. Overall, lineage L1 was the absolutely dominant strain, accounting for 83.23% (561/674) of all sequences, followed by L5 (11.72%), L8 (4.15%), and L3 (0.89%). Within L1, sub-lineage L1C was predominant, representing 81.82% of the L1 sequences. From 2019 to 2024, the detection rate of L8E showed an overall downward trend. Compared with 2019, the proportion of L5A in 2024 increased markedly. L3 was only detected during 2020–2022 and at a low proportion. The detection rate of L1A fluctuated considerably over the years, falling below 5% in 2020–2021 but rising to 24.67% in 2024. The proportion of L1C has fluctuated since 2020, accounting for 64% in 2024. Among the provinces surveyed, L1C was the dominant sub-lineage in most regions, whereas L1A was the predominant sub-lineage only in Anhui Province.

Gene mutation and recombination are critical driving forces shaping the genomic and amino acid homology of PRRSV, and they exert substantial effects on viral genome evolution and pathogenicity (Stadejek et al., 2006; Liu et al., 2025). In a 2024 study analyzing PRRSV ORF5 genes from five provinces in northern China, Jian et al. reported that the nucleotide identities among detected strains were 82–90% between lineages and 90.9–99.3% within lineages (Jian et al., 2025). In a global classification of 82,237 PRRSV ORF5 sequences into lineages L1–L11, inter-lineage pairwise nucleotide distances of PRRSV-2 were typically greater than 11%, whereas intra-lineage distances were generally less than 11% (Yim-Im et al., 2023). In the present study, the nucleotide sequence identities of 674 PRRSV ORF5 genes ranged from 75.3 to 100%, and the amino acid sequence identities ranged from 67.7 to 100%. The wide range of sequence identities observed from 2020 to 2022 was associated with the co-circulation of multiple sub-lineages, including L1A, L1C, L3, L5A, and L8E, which contributed to the reduced homology during this period. Similarly, from 2023 to 2024, the increased dominance of lineage L1 was accompanied by a significant rise in nucleotide and amino acid homology. Intra-sub-lineage homology analysis of ORF5 sequences revealed relatively high divergence within L1C, which may be related to the large number of L1C samples included in this study.

The ORF5 gene encodes several functional domains, including neutralizing epitopes, transmembrane regions, T-cell epitopes, B-cell epitopes, and a signal peptide. The GP5 protein is involved in viral replication, assembly, and the induction of neutralizing antibodies; therefore, deletions, insertions, or mutations in GP5 can alter viral replication efficiency and vaccine-induced protection (Jian et al., 2025; Luo et al., 2023; Yin et al., 2021). As shown in Table 2 and Figure 5, amino acid mutations in ORF5 were distributed across neutralizing antibody epitopes, signal peptide regions, and T-cell epitopes. These mutations provide a basis for understanding changes in viral virulence and vaccine efficacy.

PRRSV can be transmitted via aerosols, personnel, vehicles, and other fomites. Since the emergence of African swine fever in China in 2018, prevention and control strategies in Chinese swine farms have undergone profound changes. Notably, by the end of 2024, most commercial pig farms had renovated and upgraded their ventilation systems with positive-pressure air filtration, accompanied by reduced PRRSV vaccine usage. Meanwhile, the concept of PRRSV eradication and the establishment of PRRSV double-negative (nucleic acid-negative and antibody-negative) breeding herds have been widely proposed at academic conferences. It is hypothesized that these industry-wide changes in biosecurity and prevention measures may reshape the epidemiological landscape of PRRSV in China, yet the data from the present study are insufficient to verify this causal correlation due to the lack of control variables for biosecurity levels and quantitative statistical analysis models. Continuous molecular surveillance of PRRSV is essential to preliminarily explore the potential association between these control strategies and viral epidemic trends, and future research is required to validate the above hypothesis through rigorous experimental design: specifically, setting up comparative cohorts of pig farms with different biosecurity levels (e.g., farms with/without positive-pressure air filtration systems) and constructing statistical models to quantitatively analyze the correlation between biosecurity interventions and fluctuations in the proportions of major PRRSV lineages (e.g., L1C and L1A). Such follow-up studies will provide a more robust scientific basis for the integrated prevention and control of PRRSV.

## 5 Conclusion

In conclusion, 674 PRRSV ORF5 sequences collected from 25 provinces in China between 2019 and 2024 were analyzed. These sequences were classified into lineages L1, L3, L5, and L8, among which L1 was the predominant lineage. Sub-lineage epidemiological analysis showed that L1C was the dominant strain in most of the surveyed provinces. Although L1C remained dominant, its detection rate exhibited an initial increase, followed by a decline and a subsequent rebound. The detection rate of L1A fluctuated markedly but showed an overall upward trend. The detection rate of L5A displayed a fluctuating increase, whereas that of L8E showed a fluctuating decrease. The detection rate of L3 remained extremely low. Regarding nucleotide and amino acid identities, values were relatively lower from 2020 to 2022 and higher from 2023 to 2024. L1C exhibited the lowest sequence identity among the sub-lineages, while the other sub-lineages showed relatively higher identities. Complex amino acid mutations were observed at multiple sites that may influence the biological characteristics of PRRSV. Collectively, these findings provide a valuable basis for informing the prevention and control of PRRSV in China.

## Data Availability

The datasets presented in this study can be found in online repositories. The names of the repository/repositories and accession number(s) can be found in the article/Supplementary material.

## Footnotes

1
https://www.ncbi.nlm.nih.gov/

2
https://itol.embl.de/
